# Supplementary material for: Mental health challenges among adolescents and young adults with perinatally acquired HIV: Key findings from the I’mPossible program in India
Source: PLOS Ment Health. 2025 Aug 1;2(8):e0000396. doi: 10.1371/journal.pmen.0000396 (PMC12798371; doi:10.1371/journal.pmen.0000396)
Supplement: Attachment — Submitted filename: Response to Comments_Mental Health IF_2July2025-1 (1).docx [file pmen.0000396.s004.docx]

**Reviewer #1:**

***Lines 95-96: Statement could suggest that double burden exists as a function of being a LMIC, whereas I believe the intention is to indicate that this is where the bulk of the affected population with APHIV lives.***

Response: To clarify our intended meaning and avoid implying causality, we have revised the sentence to read: “While ART has contributed to decreased HIV-related mortality overall,²⁷–²9 the double burden of the HIV epidemic and common mental disorders (CMD) is poorly recognized or addressed in low- and middle-income countries (LMICs), where approximately 90% of adolescents and young people living with HIV (APHIV) reside.” The wording better emphasizes the high concentration of the affected population in LMICS.

***Lines 104-109 - missed substantive review of evidence: Bhana, A., et al. (2020). "Mental health interventions for adolescents living with HIV or affected by HIV in low- and middle-income countries: systematic review." BJPsych Open 6(5): e104.***

Response: We have incorporated the Bhana et al. (2020) reference in our discussion of mental health interventions for adolescents living with or affected by HIV in LMICs.

***Lines 109-110: Are there no studies in India or similar to India's context?***

Response: There are studies on mental health for adolescents, and also studies describing mental health among people living with HIV in India which we have referenced. However we have not found any studies examining mental health interventions specifically tailored for APHIV in India.

***Lines 114-133: Description of I'm Possible Fellowship should clarify who ultimately became the participants of this study and who are the peers. How this the program interact with the child care institutions. These details appear to be in another publication, but it would be useful to provide a clearer statement regarding these aspects here. In short the section detailing participants and study setting should be rewritten to provide clarity and appropriate detail for a reader who is unfamiliar with previous work. Given that there appear to be several groups recruited for various purposes, it might be helpful to detail these in a flow chart that details these - otherwise its difficult to keep track of it!***

Response: A clearer description of the I’mPossible Fellowship has been added to clarify the study participants, distinguish between Fellows and peers, and explain how the program engages with childcare institutions. It now reads as follows:

“The I’mPossible Fellowship program was established in the southern states of Karnataka and Tamil Nadu through collaborative efforts with child care institutions (CCIs) and community-based organizations providing integral support, such as safe residential care, medical treatment, education, and psychosocial assistance, to children and APHIV in institutional and family-based care, ages 8-26 years (peers). Trained youth living with HIV, ages 18-27 years (fellows), provide this support for peers through one-on-one sessions and group sessions over a 48-week period, in the critical domains of health, education, livelihood, and gender. Fellows often reside in partner CCIs or maintain regular contact with peers in family-based care through phone calls. Their role also includes monitoring ART adherence and addressing a range of challenges peers may face, including academic difficulties, emotional distress, or barriers in accessing healthcare services.”

To clarify participants and peers, we now clearly describe the participants in this study as a subset of peers within the I’mPossible Fellowship program as follows: “Eligible participants in this study were peers of the program, between ages 15-24 years, received a diagnosis of HIV prior to 10 years of age, were resident in CCIs or in family-based care.”


***Line 220: The comment that data saturation was reached for all major topics appears to belong in the analysis section.***

Response: The sentence “Data saturation was reached for all major topics.” has been moved to the *Insights from focus group discussions* section in line 341.

***Lines 228-231: While determining what constitutes low resilience is context dependent, is there any basis for the percentile cut offs determined for this study - either in the literature or similar studies elsewhere?***

**Response:** The CYRM-R is a remarkable tool for measuring resilience adapted for youth, however the tool does not have universally established clinical cut-offs. Resilience tends to vary widely in different contexts rendering a fixed threshold to be impractical. Developers of the tool from the Resilience Research Centre recommend that high and low scorers are contrasted within the sample. Following these guidelines, we divided the scores into quartiles and picked the lowest quartile (scores below the 25th percentile of all scores) to be defined as “low resilience”. This method is supported in the broader literature as a reasonable way to operationalize lower resilience in non-clinical, population-based samples. (Ref: Resilience Research Centre. (2022). CYRM and ARM user manual v2.5. Halifax, NS: Resilience Research Centre, Dalhousie University. Available at: <http://www.resilienceresearch.org/>).

This explanation is now added to the Methods section for added clarity. Additionally, as noted, although the 25^th^ percentile threshold is a selection by the investigator team, this is also why we chose to pursue sensitivity analysis to determine if using different percentile cutoff thresholds materially changed any results.

***Line 231-234: It is unclear why the cut off for the PHQ-9 and GAD-7 are set at such a low value. Can the author's reference validation studies in India that indicate these to be appropriate as typically a cut off of >=10 is used for both PHQ-9 and GAD-7. I note however that in the results section, description of scores that are >=10 are also presented.***

Response: We recognize that a cut off of >=10 for both PHQ-9 and GAD-7 is generally considered as a positive screen for “clinically significant” depression and anxiety. However, the decision to define positive screens for depression and anxiety as PHQ-9 and GAD-7 scores in the "mild or above" severity category (i.e., scores ≥5) in our study was informed by prior community-based research among adolescents in India, which has employed similar cut-offs for initial screening. For example, in the study *Patient Health Questionnaire-9 as an effective tool for screening of depression among Indian adolescents*, a PHQ-9 cut-off score of was found to be optimal, with a sensitivity of 87.1% and specificity of 79.7% (Ganguly et al., 2013). Similarly, Singh et al. (2017), in their study on adolescents in Chandigarh, used a cut-off of ≥5 for the PHQ-9 to identify depressive symptoms. An additional Indian study focusing on individuals with type 2 diabetes also used the mild symptom threshold (i.e., ≥5) to evaluate improvements in GAD-7 and PHQ-9 scores (Tripathi et al., 2024).

Furthermore, our rationale for including individuals with scores ≥5 aligns with existing evidence suggesting that individuals with mild symptoms are at increased risk of progression to moderate or severe illness. For instance, guidelines from the National Institute for Health and Care Excellence (NICE) recommend augmented peer support and monitoring for individuals with mild depression/anxiety, recognizing the likelihood of symptom progression (National Collaborating Centre for Mental Health, 2011). This is particularly relevant to our population since the I’mPossible Fellowship is fundamentally a peer support intervention. Hence identification of peers with positive screens with cut offs ≥5 enables us to identify additional ways to augment existing peer support as a protective intervention for progression to more severe CMD. Additionally, a systematic review by Zhang et al. (2023) found that subthreshold depression could be a significant predictor of developing major depressive disorder.

1. Ganguly S, Samanta M, Roy P, Chatterjee S, Kaplan DW, Basu B. Patient health questionnaire-9 as an effective tool for screening of depression among Indian adolescents. J Adolesc Health. 2013 May;52(5):546-51. doi: 10.1016/j.jadohealth.2012.09.012. Epub 2012 Dec 2. PMID: 23299020.
2. National Collaborating Centre for Mental Health (UK). Common Mental Health Disorders: Identification and Pathways to Care. Leicester (UK): British Psychological Society (UK); 2011. PMID: 22536621.
3. Singh MM, Gupta M, Grover S. Prevalence & factors associated with depression among schoolgoing adolescents in Chandigarh, north India. Indian J Med Res. 2017 Aug;146(2):205-215. doi: 10.4103/ijmr.IJMR_1339_15. PMID: 29265021; PMCID: PMC5761030.
4. Tripathi P, Sharma B, Kadam N, Tiwari D, Kathrikolly T, Vyawahare A, Biswas MD, Vijayakumar V, Kuppusamy M, Ganla M, Saboo B. Improvement in symptoms of anxiety and depression in individuals with type 2 diabetes: retrospective analysis of an intensive lifestyle modification program. BMC Psychiatry. 2024 Oct 22;24(1):714. doi: 10.1186/s12888-024-06130-2. PMID: 39438811; PMCID: PMC11520180.
5. Zhang R, Peng X, Song X, Long J, Wang C, Zhang C, Huang R, Lee TMC. The prevalence and risk of developing major depression among individuals with subthreshold depression in the general population. Psychol Med. 2023 Jun;53(8):3611-3620. doi: 10.1017/S0033291722000241. Epub 2022 Feb 14. PMID: 35156595; PMCID: PMC10277767.

We have included the rationale for choosing the ≥5 cut off in the data definitions section for clarity:

“The presence of a positive screen for depression was defined as a severity of mild and above (score of ≥5) according to the PHQ-9,^52^ and presence of a positive screen for GAD was defined as a severity of mild and above (score of ≥5) based on the GAD-7.^61^ These cut-offs were chosen for several reasons: (i) Prior community-based studies in India among adolescents in India have utilized similar cut-odds for initial screening of CMD,^62–64^ (ii) Existing evidence suggests that individuals with mild depression and/or anxiety are at risk of progression to moderate or severe illness with estimates ranging from 2% to 45%.^65–67^ Therefore, among adolescents, guidelines suggest close monitoring as well as augmented supports, including peer support for individuals who have positive screens for mild depression and/or anxiety.^68^ This is particularly relevant for our population of peers within the context of the I’mPossible Fellowship as identification of peers with positive screens for mild depression and/or anxiety in addition to those with positive screens for more severe depression and/or anxiety enables us to identify additional ways to augment existing support for these peers.”

***General Comment on Analysis: It may be more useful to only undertake an analysis using those scoring >=10 rather than those who scored in the mild category (often regarded as transient and self-correcting). It may also be useful to separate the analysis of those living in CCIs relative to families. While it may reduce the sample size for the respective analysis, it could provide a clearer picture of the findings. This would potentially avoid the problem of spurious positive findings. My endorsement of the statement that the analytic methods are appropriate does not take account of the above suggestion.***

Response: We agree with the reviewer that examining moderate and above categories of CMD (score ≥10 for depression and GAD) may yield useful findings. We re-analysed our data with these higher scores but found that univariate and multivariate analyses yielded similar results to the lower score cutoff of ≥5, with no additional significant associations. This is now stated in the revised manuscript. As explained in the above paragraph, we chose to present the overall analysis using PHQ-9 and GAD-7 scoring ≥5 as we believe that the “mild’ category for depression and anxiety symptoms are important to recognize among this population.

We also agree that disaggregating by living situation (i.e., CCIs vs. family care) could offer additional insights, and we conducted cross-tabulations to explore this and did not find a significant difference. We chose not to disaggregate the main analyses primarily due to the limited sample size; our overall sample size is 185 and disaggregating this would reduce statistical power further, limit the number of covariates that can be included and increase the risk of unstable estimates in multivariable regression models, our primary analysis.

***Lines 323-337: Suggesting an association exists on the basis of directionality (not significant) is not warranted.***

Response: Our intention was not to imply an association based solely on directionality without statistical significance; hence we have removed all the sentences that refer to directionality. The revised paragraph now reads as:

“In multivariable analysis of a positive screen for GAD (severity of mild and above; GAD-7 score of ≥ 5), double orphan status emerged as a significant correlate (aOR: 2.10, 95% CI: 1.07-4.09, p=0.030) associated with a two-fold increase in the odds of having a positive screen for GAD (Table 3). In the univariable and multivariable analysis of a positive screen for depression, no correlates showed statistically significant associations. (Table 3). When at least one CMD (depression and/or GAD) was considered, none of the correlates were statistically significant in their association with having a positive screen for at least one CMD. Low resilience (using thresholds of ≤ 15^th^, ≤ 33^rd^, and ≤ 50^th^ percentiles) in separate models did not materially change these findings”.

***Lines 507-520 - this description is not part of results and should be in recommendations section***

Response: Including the discussion of task-shifted, stepped-care approaches here is important because it directly connects the study’s findings on the distinct challenges APHIV face in school and work settings, such as exclusion and disclosure concerns, with potential alternative models for delivering mental health interventions. This explanation helps illustrate how evidence-based, peer-led, and youth-friendly strategies could address identified barriers, providing a critical link between the results and implications for care delivery. This section is now added in the Discussion section of the revised manuscript.

***Lines 455: The increased odds of positive screens for anxiety only occur if mild anxiety is included, but not anxiety at more severe levels. The argument about directionality is possibly overstating the findings and greater caution is required to avoid over interpreting non-significant findings.***

Response: We have revised the sentence to: “We found that double orphan status was significantly associated with increased odds of positive screens for anxiety, consistent with other studies in which bereavement has been found to be significantly associated with CMD among young people with HIV. Although other structural and psychosocial vulnerabilities, such as school discontinuation and low resilience were not statistically significant in our study, these factors have been significantly associated with CMD in other global studies among young people with HIV. Additionally, although viral suppression was not significantly associated with CMD in our study, studies across several settings also suggest that APHIV who struggle with viral suppression or are less engaged in HIV care may be at greater risk for experiencing moderate to severe CMD”. This revision aims to remove overstatements about directionality and more accurately reflects the limitations of the findings.

***Lines 497-505: Given that no significant associations were noted for CMD and stigma, what would be the basis for this concluding statement? It is generally overstating the relationship which is potentially likely but not demonstrated in this study!***

Response: Although no significant associations were noted between CMD and stigma in the multivariable models, our qualitative exploration did illuminate experiences of internal versus external stigma and the contribution of external stigma in the experience of symptoms of anxiety and/or depression among peers. We have therefore rephrased the statement to: “The qualitative narratives highlight how external stigma contributes to CMD vulnerabilities among APHIV, underscoring the need for CMD interventions to be stigma-informed while also tailored to the distinct needs of sub-groups, such as orphans and those growing up in CCIs.

***Lines 513-514: As far as I can tell, task shifting was not measured in this study.***

Response: Although task-shifting was not explicitly measured in this study, the paragraph discussing task-shifted, stepped-care approaches is intended to contextualize the potential implications of peer-led support. It highlights that school-based interventions and HIV clinics may not be appropriate settings for mental health interventions, given our findings that school exclusion and disclosure are significant concerns for APHIV.
 ***Lines 528-530: None of the elements described in this sentence was explicitly measured. For example "proof of concept that with partnership and investment, APHIV can not only provide peer support but also be trained to conduct screening for CMD among their peers. This use of peers for CMD does not demonstrate efficacy as an approach compared to other approaches.***

Response: Our intention was not to present this as definitive “proof of concept,” but rather to describe the feasibility and acceptability of engaging trained APHIV in this role within a community-led research context. To avoid overstating our findings, we have revised the sentence to reflect the exploratory nature of this work and clarified that the study provides preliminary insights into potential peer-delivered models, rather than an efficacy evaluation.

***Lines 569: The cut off of >=10 for PHQ-9 and GAD-7 presents only a small percentage of individuals who may be depressed, anxious or both. Is this burden similar to that of depression and anxiety in the general population or in relation to youth who are orphaned but who are not HIV positive. The concluding statement should be more nuanced to reflect these complexities.***

Response: External comparison is challenging given the high variability in both measures and prevalence of depression and anxiety across different adolescent populations (school going versus non-school going, rural versus urban, younger versus older adolescents) in different states in India (Rajkumar et al., 2022; Malhotra et al., 2014). However, specific to depression, we were able to compare our estimates to studies among school-going adolescents in a similar age band in a rural district with geographic overlap, where PHQ-9 was used for depression screening. We found a much higher proportion in our cohort who screened positive for both mild depression (41.1% versus 28.3%) as well as moderate and above depression (14.6% versus 10.7%). We also found one study evaluating depression among adolescents in a similar age band with non-HIV chronic illnesses, albeit in a different state in India (Mohta et al., 2024). This study similarly used a PHQ-9 cut off of ≥5. Again, we found our estimates of positive screening for depression to be higher (55.7% versus 42.8%). This is now included in the discussion section.

Nevertheless, we recognize the need for a more nuanced conclusion given the challenges with external comparison and have therefore revised the conclusion to this:

We conclude that immediate and long-term investment to integrate mental health screening and intervention implementation across the pediatric-to-adult HIV care continuum is needed for all adolescents with HIV in India, though future research should critically examine the mental health needs of those who are under-engaged in care and/or are not virally suppressed. Implementation research that incorporates community-based participatory research principles is needed to inform both screening and delivery of evidence-based interventions for CMD among APHIV. Such investments need to be a high priority as they have the potential to not only shape HIV care outcomes but also yield lasting benefits beyond HIV across the life-course for young people with HIV.

1. Malhotra S, Patra BN. Prevalence of child and adolescent psychiatric disorders in India: a systematic review and meta-analysis. Child Adolesc Psychiatry Ment Health. 2014 Jul 21;8:22. doi: 10.1186/1753-2000-8-22. Erratum in: Child Adolesc Psychiatry Ment Health. 2017 Jul 12;11:39. doi: 10.1186/s13034-017-0175-2. PMID: 25071865; PMCID: PMC4113132.
2. Mohta A, Malhotra S, Kalaivani M, Patra BN, Nongkynrih B. Chronic Illnesses and Depression among Community-based Adolescents in Rural Haryana, India. Indian J Public Health. 2024 Apr 1;68(2):287-290. doi: 10.4103/ijph.ijph_327_23. Epub 2024 Jun 29. PMID: 38953819.
3. Rajkumar E, Julia GJ, Sri Lakshmi K NV, Ranjana PK, Manjima M, Devi RR, Rukmini D, Christina G, Romate J, Allen JG, Abraham J, Jacob AM. Prevalence of mental health problems among rural adolescents in India: A systematic review and meta-analysis. Sci Rep. 2022 Oct 4;12(1):16573. doi: 10.1038/s41598-022-19731-2. PMID: 36195719; PMCID: PMC9532445.

***There are minor grammatical issues scattered throughout the MS. For example, Lines 144-146: Participants... Fellowship comprising 257 peers. Those aged 13 years...study.***

Response: The manuscript has been reviewed and edited to address grammatical issues.

**Reviewer #2:**

***Lines 315 and 316: The font size for the table label seems smaller than that of table 1. Please note for consistency.***

Response: The tables have all been reviewed and edited to ensure consistent formatting.

***Lines 431-437: The first paragraph of the ‘Discussion section’ should highlight the main findings of your research. Your current write-up speaks to the aim and objectives of your work, but the key findings not explicitly stated. This is a section of your article a reader to check to see the main findings of your work. Consider revising.***

Response: We have explicitly stated the main findings and have consolidated the first paragraph with the second paragraph so that the reader can immediately check the key takeaways of our study.

***Lines 464-466 “One may anticipate that APHIV experiencing challenges with viral suppression and engagement in HIV care will have an even higher burden of moderate and above CMD severity”. Please review this statement for clarity. It sounds vague.***

Response: The sentence has been edited for clarity and now reads: “APHIV who struggle with viral suppression or are less engaged in HIV care may be at greater risk for experiencing moderate to severe CMD.”

***Line 473: Insert a comma after ‘others’***

Response: Comma has been added after ‘others’.

***Lines 471-475: “In multivariable models, although there was no significant difference in association of positive screens for CMD between CCI-based APHIV versus others possibly owing to small sample sizes, qualitative exploration yielded narratives that highlight divergence around mental health stressors in these sub-groups”. This sentence seems long. Consider breaking it into easy-to-read sections.***

Response: The sentence has been broken down into two, and now reads: “In multivariable models, there was no significant difference in association of positive screens for CMD between CCI-based APHIV versus others, possibly owing to small sample sizes. However, qualitative exploration yielded narratives that highlight divergence around mental health stressors in these sub-groups.”

***Lines: 477-480: “Consistent with other studies, APHIV in CCIs received minimal family support and described strained relationships with extended family members as a source of stress, whereas for APHIV living with families, a parent with a shared HIV status, as well as other family members were sources of support, in addition to peers”. This sentence is long. Consider breaking it.***

Response: The sentence has been broken apart into two and now reads: “Consistent with other studies, APHIV in CCIs received minimal family support and described strained relationships with extended family members as a source of stress. In contrast, for APHIV living with families, a parent with a shared HIV status, as well as other family members were sources of support, in addition to peers.”

***Lines 516-520: “While high client volume at public sector ART centers may pose challenges to integrating task-shifted roles for mental health care delivery, youth-friendly models of HIV care led by trained youth and community members show promise globally, 101–104 and in Indian settings105 in addressing CMD among APHIV”. This sentence structure is not well written. Kindly revise it. Also the sentence is long which could it difficult for reader to follow through. In general, please try and avoid long sentences in the article.***

Response: The sentence in question has been edited for clarity, and the manuscript has been revised to avoid long sentences.

**Line 524: Please change ‘are’ to ’were’. It should read in past tense.**

Response: Thank you for your suggestion. However, we intended this sentence to reflect the current status of peer supporter integration in national HIV programs. As such, we have retained the present tense “are” to accurately convey that peer supporters continue to be part of these programs in countries like Zimbabwe.

***Line 551: …higher than what?...The comparison sounds incomplete. Kindly revise***

Response: We have clarified that the comparison of CMD prevalence in India was between women and men, and this is now stated more explicitly in the revised text.
